# Cerebral responses to famous face recognition as a potential functional biomarker of mild cognitive impairment

**DOI:** 10.3389/fnins.2025.1698395

**Published:** 2026-01-16

**Authors:** Michihiko Koeda, Yumiko Ikeda, Yoshiro Okubo, Amane Tateno

**Affiliations:** 1 Department of Neuropsychiatry, Nippon Medical School Tama Nagayama Hospital, Tokyo, Japan; 2 Department of Neuropsychiatry, Graduate School of Medicine, Nippon Medical School, Tokyo, Japan; 3 Department of Pharmacology, Graduate School of Medicine, Nippon Medical School, Tokyo, Japan

**Keywords:** biomarker, *famous* faces, fMRI, *mild cognitive im*pairment, *parahippocampal gy*rus

## Abstract

**Background:**

Social cognition impairments—including difficulties in recognizing personally familiar faces—occur early in mild cognitive impairment (MCI) and can lead to social withdrawal, reduced motivation, and secondary depression. Face recognition is central to social cognition, yet its neural basis in MCI remains insufficiently understood. This study examined whether task-based fMRI during famous face recognition could capture early alterations in the parahippocampal gyrus (PHG) and posterior cingulate cortex (PCC), key nodes supporting semantic access and internally directed cognition within the default mode network (DMN).

**Methods:**

Thirty-two participants (20 healthy controls, 12 MCI) completed two fMRI tasks: famous vs. non-famous face judgment and face vs. object categorization. A 2 × 2 factorial analysis assessed Group and Task effects, and small-volume correction was applied to PHG and PCC.

**Results:**

Behavioral accuracy was comparable between groups; however, whole-brain analyses revealed markedly reduced activation in the left PHG and PCC in the MCI group during socially meaningful face processing. ROI analyses further demonstrated that the left PHG reduction remained significant after FWE correction, whereas PCC showed a weaker reduction that did not survive correction for multiple comparisons.

**Conclusion:**

These findings suggest early alterations in PHG–PCC networks that precede observable behavioral decline in MCI. In particular, reduced activation in the left PHG may reflect early disruptions in semantic access and internally directed processing. Assessing these socially relevant neural circuits alongside established amyloid and tau biomarkers may provide complementary functional insight into early cognitive vulnerability in individuals at risk for dementia.

## 1 Introduction

Early diagnosis and pathophysiological understanding of dementia require not only pathological biomarkers but also a precise evaluation of the neural substrates underlying cognitive impairments (Adolphs, 2009). In recent years, the ATN framework—based on amyloid β deposition (A), tau pathology (T), and neurodegeneration (N)—has been widely adopted in Alzheimer’s disease (AD) research, facilitating the identification of *in vivo* pathological markers (Albert et al., 2011; Aminoff et al., 2013). However, in addition to these pathological biomarkers, the investigation of functional brain changes that directly reflect the emergence of cognitive symptoms remains indispensable (Aminoff et al., 2013; Andrews-Hanna et al., 2010; Bai et al., 2009).

Among these, impairments in social cognition are known to occur even in the early stages of mild cognitive impairment (MCI) and AD (Bar et al., 2001). Such deficits often contribute to social withdrawal, loss of motivation, and secondary depression (Brodaty et al., 2013). Within social cognition, face recognition plays a pivotal role, as it forms the basis of interpersonal understanding and human relationships (Buckner et al., 2008; Dickerson et al., 2005). In advanced AD, recognition of family members’ faces is often severely impaired (Friston et al., 2011), but the neural substrates of face recognition in the MCI stage remain insufficiently elucidated (Frith and Frith, 2007). Importantly, MCI is a heterogeneous clinical syndrome that does not invariably progress to Alzheimer’s disease, and only a subset of individuals with MCI will eventually develop dementia.

Neuroimaging studies of face recognition have proposed a dual-route model consisting of the ventral stream (including the fusiform gyrus and parahippocampal gyrus, PHG) (Gobbini and Haxby, 2007; Hafkemeijer et al., 2012; Hammers et al., 2003) and the dorsal stream (including the posterior cingulate cortex, PCC, and parietal association areas) (Haxby et al., 2000; Ishai, 2008). In particular, the fusiform gyrus and PHG are crucial for person identification and semantic memory retrieval (Haxby et al., 2000; Ishai, 2008), while the PCC functions as a hub of the default mode network (DMN), supporting autobiographical memory and contextual processing (Adolphs, 2009; Jack et al., 2018; Kurth et al., 2015). Resting-state fMRI studies have demonstrated that MCI is associated with reduced functional connectivity of the DMN (Frith and Frith, 2007; Leveroni et al., 2000; Mirza et al., 2017), suggesting that DMN vulnerability may represent an early neural change associated with an increased risk of progression to Alzheimer’s disease in some individuals.

Emerging neuroimaging work has also begun to clarify how specific components of the face-processing network are affected in neurodegenerative conditions. Notably, Canário et al. (2023) reported that early Alzheimer’s disease does not show functional alterations in the fusiform face area (FFA) itself (Pievani et al., 2011), but instead exhibits changes in adjacent ventral-stream regions—most prominently the fusiform body area (FBA) and the visual word form area (VWFA). These findings suggest that early ventral-stream vulnerability may be localized to select subregions, rather than reflecting a widespread disturbance across classical face-selective cortex. Similarly, Graewe et al. (2013) observed reduced FFA activation during the processing of 3D motion-defined faces (Psychology Software Tools, Inc, 2012); however, the authors emphasized that this reduction likely reflects the dynamic, non-static nature of the stimuli, rather than a disease-specific impairment of static face perception. Together with the results of Sauer et al. (2006), this evidence indicates that although ventral-stream alterations can arise early in cognitive decline, the affected regions and their underlying mechanisms vary considerably across studies and depend strongly on stimulus properties and task demands (Raichle et al., 2001). Given these findings, a key outstanding question is whether individuals with mild cognitive impairment (MCI) exhibit functional alterations in medial temporal components of the ventral stream—particularly the parahippocampal gyrus—as well as in default mode network (DMN) regions known to be vulnerable in early MCI, specifically during the recognition of familiar faces presented as static images.

Moreover, the recognition of famous and personally familiar faces represents a prototypical form of socially meaningful processing (Ramon et al., 2011; Ranganath and Ritchey, 2012; Rossion et al., 2003), and previous fMRI studies have consistently shown the involvement of PHG and PCC (Buckner et al., 2008; Schaefer et al., 2018; Seeley et al., 2009). Leveroni et al. demonstrated strong left PHG activation during famous face recognition (Sperling et al., 2011), while Seeley et al. (2009) and Buckner et al. (2008) further linked this activity to semantic memory processing. These findings highlight the PHG as a central node for the recognition of socially meaningful faces.

In recent years, growing attention has been paid to the use of fMRI in assessing social cognition and non-verbal cue processing as potential early biomarkers of MCI and AD (Ramon et al., 2011; Spreng et al., 2009). A previous research reported selective impairments in social stimulus processing in neurodegenerative diseases (Sugiura et al., 2001), another research demonstrated reduced brain activation during social cognition tasks in MCI (Friston et al., 2011), and the recent study showed that performance on non-verbal recognition tasks could predict AD progression (Tsukiura et al., 2002). These findings emphasize the clinical relevance of fMRI-based evaluation of non-verbal recognition.

Because group differences in behavioral performance are often subtle or absent (Albert et al., 2011), functional imaging of socially meaningful recognition provides valuable information about the underlying neural mechanisms. In particular, famous-face paradigms reflect non-verbal and socially meaningful cognition, making them a valuable approach for examining early functional alterations associated with MCI.

Based on this background, the present study aimed to compare brain activation during famous face recognition between MCI and healthy control (HC) participants, focusing on functional changes in the left PHG and PCC as central nodes of the DMN. We also sought to clarify whether dysfunction in these regions could serve as a functional biomarker for early dementia using a 2 × 2 factorial fMRI paradigm. We hypothesized that, although behavioral performance might remain intact, MCI would show altered activation in these regions—potentially reduced due to DMN vulnerability, despite *a priori* consideration of compensatory enhancement in the PHG.

## 2 Materials and methods

### 2.1 Participants

Thirty-two individuals took part in the study: twenty healthy older adults (HC) and 12 patients with mild cognitive impairment (MCI). Diagnoses were established by experienced board-certified psychiatrists specializing in cognitive disorders and dementia assessment, using standardized clinical interviews, neuropsychological testing, and collateral information from family members. All MCI patients met diagnostic criteria for mild cognitive impairment according to ICD-10 and DSM-5, corresponding to a Clinical Dementia Rating (CDR) of 0.5–1, which requires subjective complaints, objective cognitive impairment, preserved daily functioning, and absence of dementia. No amyloid or tau biomarkers, or cerebrospinal fluid analyses, were available to determine underlying Alzheimer’s pathology. Therefore, we interpreted MCI as a clinical risk state rather than biologically defined prodromal AD, and our results should be understood at the level of the MCI syndrome.

The healthy control (HC) group consisted of cognitively healthy older adults with no history of neurological or psychiatric disorders, all of whom had a Clinical Dementia Rating (CDR) of 0. All participants were also confirmed to have no history of head trauma, alcohol dependence, or substance abuse. Group characteristics (age, sex, MMSE) followed the demographics summarized in Table 1. All participants provided written informed consent, and the study was approved by the Ethics Committee of Nippon Medical School (Approval No. 24-12-267).

**TABLE 1:** Demographic and neuropsychological characteristics of HC and MCI groups.

Variable	HC	MCI	Test	*P*-value
Gender			χ^2^(df = 1) = 8.04	<0.01
Male	15 (75.00%)	2 (16.67%)		
Female	5 (25.00%)	10 (83.33%)		
Age (years)	73.85 ± 3.17	79.25 ± 4.58	–3.60 (df = 17.4)	<0.01
MMSE (points)	29.30 ± 0.98	23.00 ± 2.83	7.45 (df = 12.6)	<0.01

HC, healthy controls; MCI, mild cognitive impairment. Values are mean ± SD for continuous variables and n (%) for categorical variables. Sex tested by chi-square; Age and MMSE by Welch’s *t*-test. All values rounded to 2 decimals.

### 2.2 Experimental design and tasks

**FIGURE 1:**
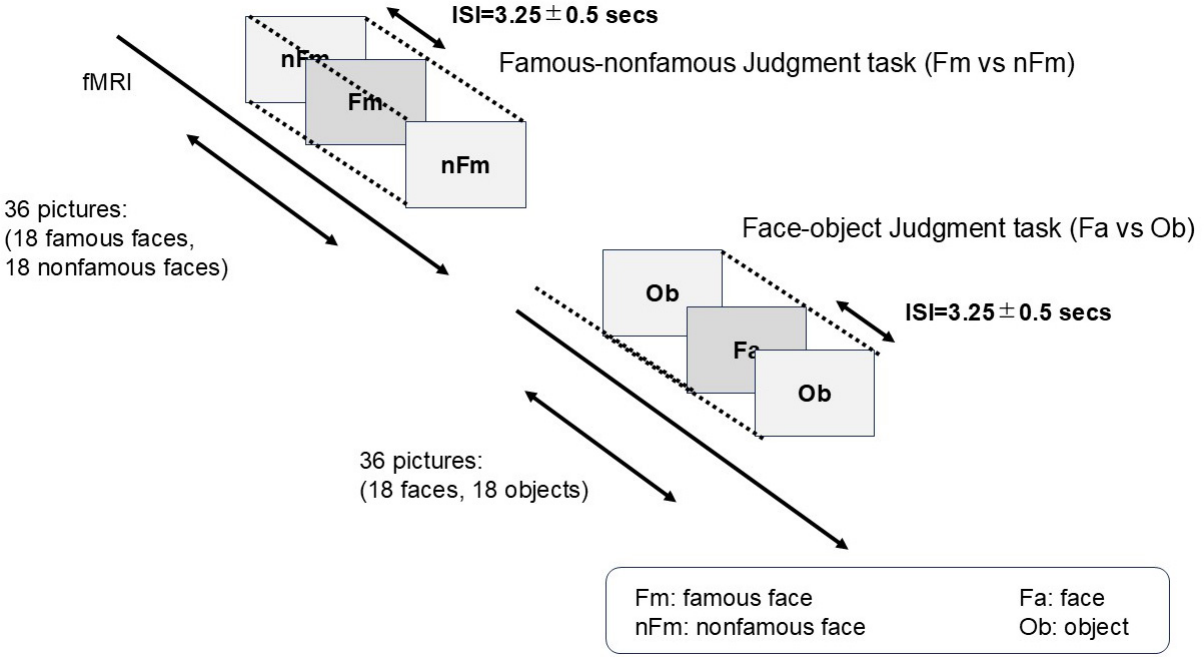
Experimental paradigm of the fMRI tasks. Participants performed two event-related tasks: (i) famous vs. non-famous face judgment, and (ii) face vs. object categorization.

Stimuli were presented using E-Prime 2.0 (Ubaldi and Fairhall, 2021). Participants performed two event-related tasks: (i) a famous–non-famous face judgment and (ii) a face–object categorization task (Figure 1). Each stimulus was displayed for 2,000 ms, followed by a jittered inter-stimulus interval (ISI) with a mean of 3,250 (± 500 ms). Responses were collected via button press, and accuracy and reaction times were recorded. These two task types constitute the Task factor used at the group level (2 × 2 full factorial design: Group × Task).

### 2.3 Image acquisition

MRI scanning was performed on a 1.5-T Philips clinical scanner. Functional images were acquired with a T2*-weighted gradient-echo EPI sequence (TR = 2,500 ms, TE = 30 ms, flip angle = 80°, FOV = 256 × 256 mm, matrix = 64 × 64, slice thickness = 4 mm, 35 contiguous axial slices). High-resolution T1-weighted structural images (MPRAGE) were also obtained (TR = 9.3 ms, TE = 4.6 ms, flip angle = 8°, FOV = 250 mm, matrix = 256 × 256, slice thickness = 1.2 mm, 160 slices).

### 2.4 Volume counts and analysis policy

The task paradigm and imaging parameters were identical across participants, but the number of acquired volumes varied because of individual tolerance. HC: 17 participants had 155 volumes, 1 had 335, and 1 had 340; MCI: 8 participants had 155, 1 had 340, 1 had 145, and 2 participants completed 78 × 2 sessions. For consistency, analyses used 155 volumes per participant whenever available. The 145-volume case was included because the GLM robustly captured task-related activity; two-run (78 × 2) data were modeled as separate sessions and combined at the subject level.

### 2.5 Preprocessing

Data were analyzed and preprocessed using Statistical Parametric Mapping (SPM8) (Van Overwalle, 2009). Steps included slice-timing correction, realignment, coregistration of functional to structural images, normalization to MNI space via DARTEL high-dimensional warping, and spatial smoothing with an 8-mm FWHM Gaussian kernel. Normalization accuracy was visually inspected for all participants. Volumes exceeding 3 mm translation or 3° rotation were censored or modeled with one-volume “scrubbing” regressors.

### 2.6 First-level analysis

For each participant, an event-related GLM was specified with four condition regressors aligned to stimulus onset, each modeled with a fixed duration of 2 s (equal to the display time): (1) Famous-face and (2) Non-famous-face from the famous/non-famous judgment task; and (3) Face and (4) Object from the face/object categorization task. Incorrect trials were modeled as a separate regressor. Six motion parameters were included as nuisance regressors. A high-pass filter (128 s) and AR (Adolphs, 2009) temporal-autocorrelation correction were applied. For two-run datasets (e.g., 78 + 78 volumes), identical condition regressors were defined in each session with session constants and motion regressors; contrasts were estimated across sessions. Individual contrast images (e.g., Famous-face > Non-famous-face; Face > Object) were taken to the group level.

### 2.7 Second-level analysis

Group-level analyses were conducted in SPM12 using a 2 × 2 full factorial model with two main factors: Group (HC vs. MCI) and Task (famous/non-famous judgment vs. face/object categorization). Main effects of Group and Task and the Group × Task interaction were tested. Whole-brain maps were assessed at an initial threshold of voxel-wise *p* < 0.005 (uncorrected) with a cluster-extent threshold of k ≥ 100 voxels. Significance was evaluated with whole-brain FDR correction. Behavioral ANOVAs (accuracy, reaction time) paralleled the imaging design.

### 2.8 Software and versioning (transparency)

Preprocessing and first-level (single-subject) GLM estimation were performed in SPM8 (Wellcome Trust Centre for Neuroimaging). Random-effects second-level analyses were performed in SPM12. Inter-subject registration used DARTEL; individual contrast images were normalized to MNI space using participant-specific DARTEL flow fields before group analysis. The design matrices, contrasts, and smoothing kernels were identical across versions, and all inferences are based on SPM12 second-level statistics. We acknowledge minor implementation differences between SPM8 and SPM12; however, because inferences rely on within-subject contrasts carried to random-effects models, these differences are not expected to materially affect the results.

### 2.9 Regions of interest

**FIGURE 2:**
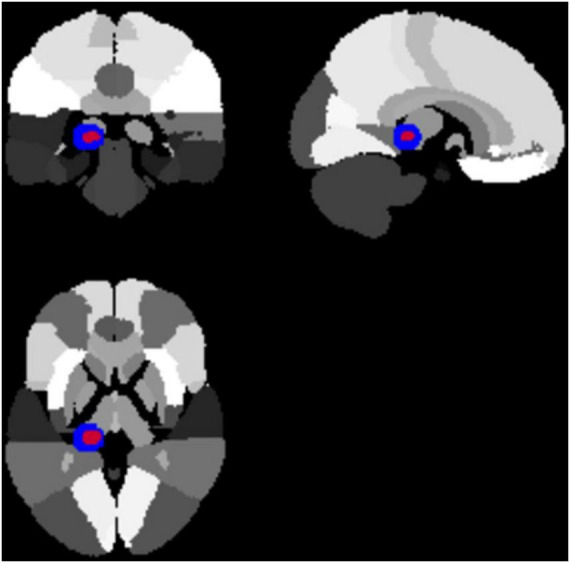
ROI analyses in PHG and PCC. The PHG ROI survived peak-level FWE correction. Group-level activation map (red) for the main effect of Group (HC > MCI) overlaid with the *a priori* defined PHG ROI [blue sphere, 10 mm radius centered at MNI (–18, –34, –8)] shown on Hammers atlas background. The spatial overlap confirms that the significant activation fell within the predefined anatomical region.

The primary ROI was the left PHG, defined *a priori* as a 10 mm radius sphere centered at MNI (–18, –34, –8), based on prior famous/personally familiar face fMRI literature (Bar et al., 2001; Leveroni et al., 2000; Sugiura et al., 2001; Tsukiura et al., 2002). The PCC [MNI (6, -54, 20)] was examined as an exploratory ROI. Small-volume correction (SVC) with peak-level FWE *p* < 0.05 was applied within each ROI. The ROI was used to test whether the peak activation for the main effect of Group would fall within this anatomically defined region. The overlap between the functional activation and the *a priori* ROI was visually confirmed using MRIcron and Hammers probabilistic atlas labeling, as shown in Figure 2.

### 2.10 Network labeling

Significant clusters were assigned to large-scale networks using the Schaefer et al. (2018) parcellation (200 parcels, 17 networks) (Visconti di Oleggio Castello et al., 2017) and cross-validated with the Hammers 95-region anatomical atlas (Yuan et al., 2021), allowing network-level interpretation (e.g., DMN subsystems) alongside anatomical labels.

## 3 Results

### 3.1 Behavioral results

Demographic and neuropsychological characteristics of the two groups are summarized in Table 1. Compared to the MCI group, the healthy control (HC) group included significantly more male participants [χ^2^(1) = 8.04, *p* < 0.01], and the HC group was significantly younger in age [*t*(17.4) = –3.60, *p* < 0.01]. In addition, MMSE scores were significantly lower in the MCI group than in the HC group [*t*(12.6) = 7.45, *p* < 0.01], consistent with their cognitive impairment. In the behavioral tasks, both groups performed with high accuracy across conditions. Two-way ANOVAs revealed no significant main effects of Group or Task, nor a significant interaction, for accuracy rates or reaction times. These findings indicate that behavioral performance was preserved in the MCI group, despite their cognitive impairment. Behavioral data are summarized in Table 2.

**TABLE 2:** Behavioral performance (AC, RT) during the two tasks by the HC and MCI group.

Domain	Measure	HC Mean ± SD	MCI Mean ± SD	Welch t (df)	*P*
AC	Fa vs. Ob	98.19 ± 3.28%	88.89 ± 27.47%	1.17 (df = 11.2)	0.27
AC	Fm vs. nFm	93.33 ± 7.77%	89.12 ± 13.79%	0.97 (df = 15.3)	0.35
RT	Fa vs. Ob	740.81 ± 112.76 msec	824.12 ± 330.22 msec	–0.84 (df = 12.6)	0.41
RT	Fm vs. nFm	1296.0 ± 216.81 msec	1276.28 ± 251.84 msec	0.23 (df = 20.6)	0.82

AC, Accuracy; RT, response time; Fm, famous face; nFm, non-famous face; Fa, face; Ob, object.

### 3.2 Main effect of group

**FIGURE 3:**
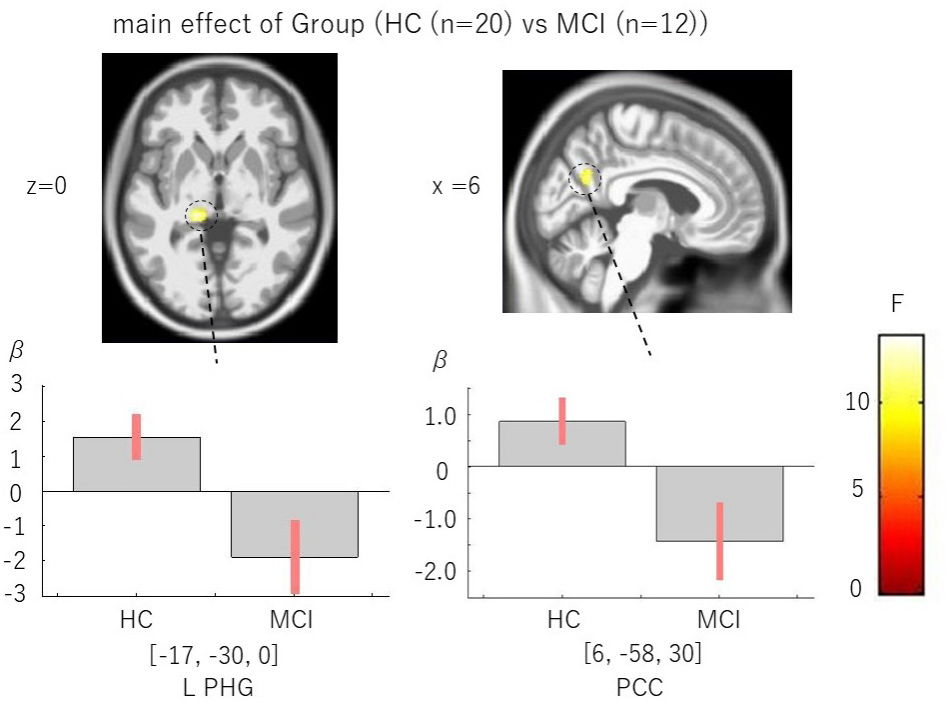
Main effect of Group (HC > MCI). Reduced activation in left PHG and PCC. Statistical parametric maps show reduced activation in MCI relative to HC, primarily in the left parahippocampal gyrus (PHG) and posterior cingulate cortex (PCC). Whole brain FDR correction of peak level was not significant. The upper panel shows the activation areas in the L PHG (left side) and PCC (right side); the lower bar graph shows beta value on the ROIs in these two regions. Maps shown at voxel-wise *p* < 0.005 (uncorrected) with a cluster-extent threshold of k ≥ 100 voxels; peak-level FDR results are indicated where applicable.

The main effect of Group revealed reduced activation in MCI compared to HC in the left PHG and right precuneus/PCC, regions central to the default mode network. These findings are illustrated in Figure 3 and summarized in Table 3.

**TABLE 3:** Main effect of group [HC (*n* = 20) vs. MCI (*n* = 12)], whole-brain results, statistical threshold: *p* < 0.005, uncorrected, k ≥ 100 voxels.

Hemishere	Brain regions	MNI coordinate	BA	ke	*F*	*Z*	*P*	FDR-corrected
L	PHG	(–17, –30, 0)	36	130	13.55	3.27	0.001	n.s.
R	Precuneus/PCC	(6, –58, 30)	7/31	177	12.1	3.09	0.001	n.s.

L, left; R, right; HC, healthy control; MCI, mild cognitive impairment.

### 3.3 Main effect of task

**FIGURE 4:**
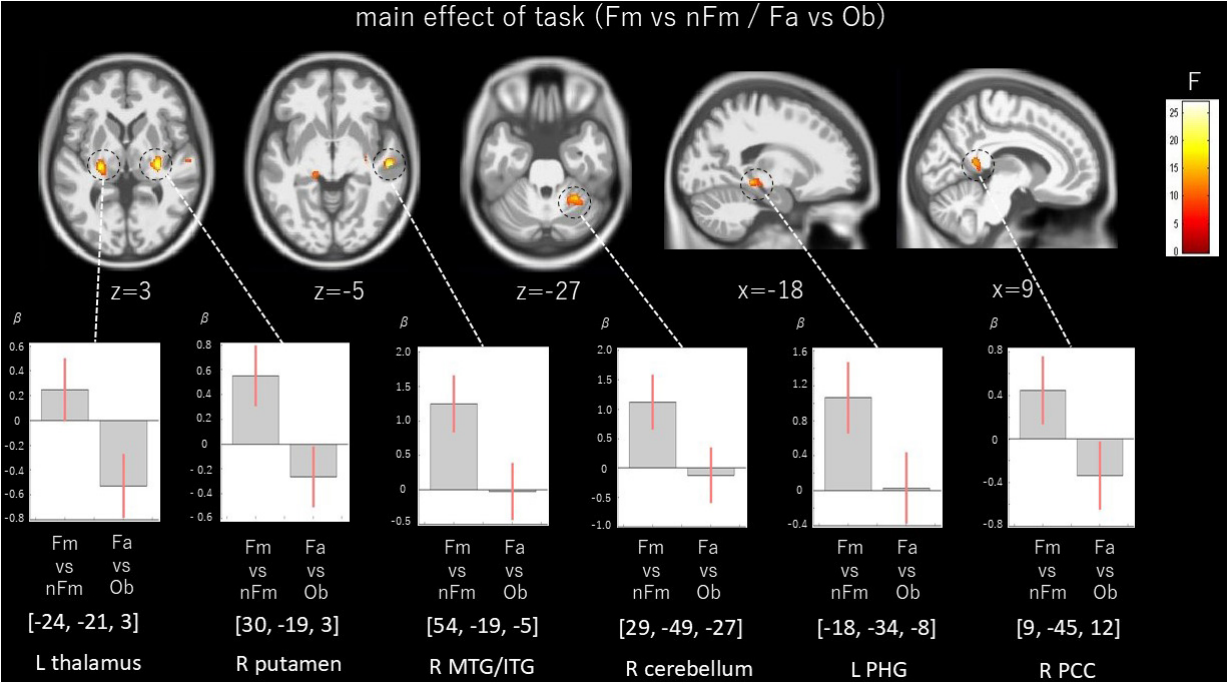
Main effect of task. Activation differences between Famous vs. non-famous judgment, and face vs. object categorization. Brain activations associated with the comparison between the Fm vs. nFm judgment task and the Fa vs. Ob categorization task, highlighting bilateral fusiform gyrus and occipital regions. Whole brain FDR correction of peak level was not significant. Fm, famous; nFm, non-famous; Fa, face; Ob, object Maps shown at voxel-wise *p* < 0.005 (uncorrected) with a cluster-extent threshold of k ≥ 100 voxels; peak-level FDR results are indicated where applicable.

The main effect of Task demonstrated significant activation differences between the famous/non-famous face judgment task and the face/object categorization task. Significant activations were found in the thalamus and PHG (BA35) in the left hemisphere, and in the putamen, middle and inferior temporal gyrus (MTG/ITG, BA21/20), cerebellum, and PCC (BA29) in the right hemisphere. These findings are illustrated in Figure 4 and summarized in Table 4.

**TABLE 4:** Whole-brain results: main effect of task (the Fm vs. nFm judgment task and the Fa vs. Ob categorization task).

Hemishere	Brain regions	MNI coordinate	BA	ke	*F*	*Z*	*P*	FDR-corrected	Network region (Schaefer)
R	Putamen	(30, –19, 3)		349	26.9	4.51	< 0.001	n.s.	SomMotB
R	MTG/ITG	(54, –19, –5)	21/20	159	23.18	4.22	< 0.001	n.s.	TempPar
L	Thalamus	(–24, –21, 3)		210	22.26	4.14	< 0.001	n.s.	
R	Cerebellum	(29, –49, –27)		389	17.05	3.66	< 0.001	n.s.	LimbicA
L	PHG	–18, –34, –8)	35	151	15.63	3.51	< 0.001	n.s.	DefaultC
R	PCC	(9, –45, 12)	29	168	15.09	3.45	< 0.001	n.s.	DefaultC

Statistical threshold of voxel-wise *p* < 0.005 uncorrected with a cluster-extent threshold of k ≥ 100 voxels. L, left; R, right; MTG, middle temporal gyrus; ITG, inferior temporal gyrus; PCC, posterior cingulate cortex; Fm, famous; nFm, non-famous; Fa, face; Ob, object.

### 3.4 Group × task interaction

**FIGURE 5:**
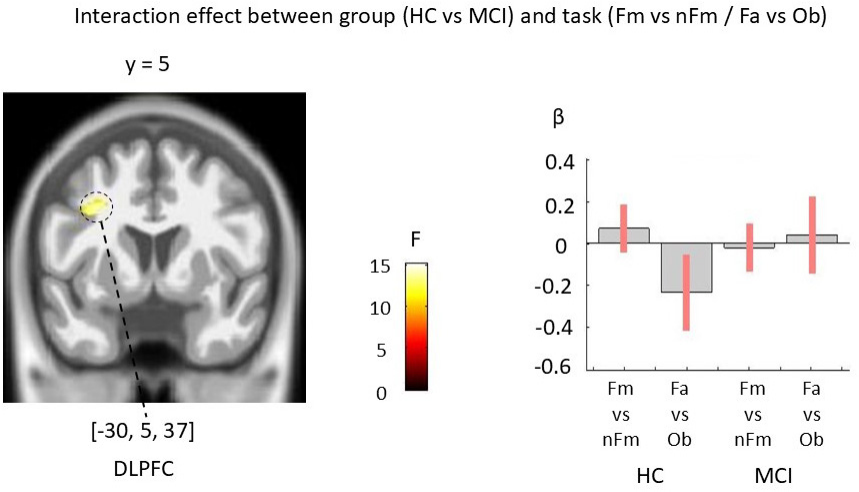
Exploratory group × task interaction effects (no cluster survived FDR correction). Exploratory statistical maps indicating differential activation patterns between HC and MCI, although no clusters survived FDR correction. Maps shown at voxel-wise *p* < 0.005 (uncorrected) with a cluster-extent threshold of k ≥ 100 voxels; peak-level FDR results are indicated where applicable.

The Group × Task interaction did not survive FDR correction at the whole-brain level. However, exploratory analyses indicated trends toward differential activation patterns at the left dorsolateral prefrontal cortex (DLPFC) between HC and MCI across the two tasks. These exploratory results are shown in Figure 5 and summarized in Table 5.

**TABLE 5:** Whole-brain results: the interaction effect between Group (HC vs. MCI) and Task (Fm-nFm vs. Fa-Ob).

Hemishere	Brain regions	MNI coordinate	BA	ke	*F*	*Z*	*P*	FDR-corrected
L	DLPFC	(–30, 5, 37)	9	206	14.68	3.4	< 0.001	n.s.

Statistical threshold of voxel-wise *p* < 0.005 uncorrected with a cluster-extent threshold of k ≥ 100 voxels. L, left; R, right; HC, healthy control; MCI, mild cognitive impairment; DLPFC, dorsolateral prefrontal cortex; Fm, famous face; nFm, non-famous face; Fa, face; Ob, object.

### 3.5 Famous versus non-famous contrasts (the Fm-nFm contrast)

**FIGURE 6:**
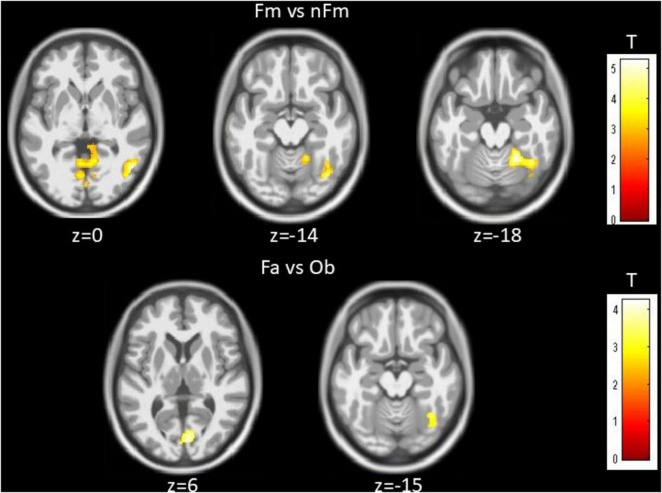
Direct contrasts for famous > non-famous and face > object across all subjects (*n* = 32). Significant activations for contrasts of the Fm vs. nFm was significantly observed at the primary visual cortex and the fusiform gyrus with the multiple comparison and Fa vs. Ob contrasts in the visual cortex and the fusiform gyrus. Maps shown at voxel-wise *p* < 0.005 (uncorrected) with a cluster-extent threshold of k ≥ 100 voxels; peak-level FDR results are indicated where applicable.

Direct contrasts between famous and non-famous face conditions across all participants revealed significant activation in the left PHG, right MTG (BA21), medial occipital cortex (MOC, BA17), right putamen, and right cerebellum. These results are illustrated in Figure 6 and summarized in Table 6.

**TABLE 6:** Whole-brain contrasts: effect of Fm vs. nFm (*n* = 32).

Hemishere	Brain regions	MNI coordinate	BA	ke	*T*	*Z*	*P*	FDR-corrected
R	Cerebellum	(23, –49, –18)		2,369	5.27	4.72	< 0.001	<0.05
R	MTG	(54, –19, –6)	21	321	5.16	4.63	< 0.001	<0.05
R	Putamen	(30,–12, 0)		589	4.44	4.08	< 0.001	<0.05
L	Parahippocampus	(–18, –34, –8)	35	553	4.33	3.99	< 0.001	<0.05
R	MOC	(6, –60, 3)	17	1,822	4.25	3.93	< 0.001	<0.05

Statistical threshold of voxel-wise *p* < 0.005 uncorrected with a cluster-extent threshold of k ≥ 100 voxels, L, left; R, right; MTG, middle temporal gyrus; Fm, famous face; nFm, non-famous face.

### 3.6 Face versus object contrasts (the Fa-Ob contrast)

The face versus object contrast (Fa > Ob), computed across all participants, revealed significant activation in the right cuneus (BA17), a core region of the early visual cortex, and the right fusiform gyrus (BA37). This result is presented in Figure 6 and summarized in Table 7.

**TABLE 7:** Whole-brain contrasts: effect of Fa vs. Ob (*n* = 32).

Hemishere	Brain regions	MNI coordinate	BA	ke	*T*	*Z*	*P*	FDR-corrected
R	Cuneus	(8, –78, 6)	17	657	4.25	3.93	< 0.001	n.s.
R	Fusiform gyrus	(38, –58, –15)	37	172	3.4	3.22	0.001	n.s.

Statistical threshold of voxel-wise *p* < 0.005 uncorrected with a cluster-extent threshold of k ≥ 100 voxels, L, left; R, right; Fa, face; Ob, object.

### 3.7 ROI analyses

ROI-based small volume correction (SVC) confirmed that the left PHG [MNI (-18, -34, -8)] showed significantly reduced activation in MCI relative to HC during famous face recognition. In addition, the right PCC [MNI (6, -54, 20)] showed a similar group-related activation difference, although this effect did not survive correction for multiple comparisons. Table 8 summarizes these statistical outcomes. These results indicate that early dysfunction of the PHG may represent a sensitive functional marker of social cognitive decline in MCI. Furthermore, the significant cluster for the main effect of Group overlapped with the *a priori* defined PHG ROI [10 mm radius centered at (-18, -34, -8)], as shown in Figure 2. This ROI-based activation survived peak-level FWE correction (*p* = 0.044), confirming anatomical and statistical convergence.

**TABLE 8:** ROI-based small volume correction (SVC) analysis on the main effect of Group (HC vs. MCI) in the left parahippocampal gyrus (PHG) and right posterior cingulate cortex (PCC).

Hemishere	Brain regions	MNI coordinate	BA	FWE-corrected	FDR-corrected	*F*	*Z*	*P*
L	PHG	(–18, –34, –8)	35	0.044	n.s.	13.55	3.27	0.001
R	PCC	(6, –54, 20)	31	n.s.	n.s.	11.71	3.04	0.001

The PHG ROI survived peak-level FWE correction, while the PCC ROI showed a subthreshold effect at the uncorrected level. L, left; R, right; PHG, parahippocampal gyrus; PCC, posterior cingulate cortex; BA, Brodmann area.

## 4 Discussion

In this study, we investigated the neural correlates of famous face recognition in MCI compared with HC. Although behavioral performance did not differ significantly between groups, fMRI analyses revealed reduced activation in MCI in key regions such as the left PHG and PCC. These results indicate that neural dysfunction in socially meaningful face recognition networks may already be present at the clinically defined MCI stage, preceding overt behavioral deficits in at least a subset of individuals who are at increased risk for progression to dementia. Rather than reflecting a deficit specific to famous-face processing, the reduced activity in PHG and PCC is better interpreted as a broader impairment in networks supporting semantic access and internally directed cognition.

### 4.1 Early vulnerability of the region of PHG and PCC

Our findings highlight the left PHG as a critical locus of dysfunction in MCI during famous face recognition (Table 8 and Figure 2). Previous fMRI studies demonstrated robust PHG activation during familiar or famous face recognition (Buckner et al., 2008; Seeley et al., 2009). The current reduction in PHG activity aligns with reports of impaired semantic memory and autobiographical recall in MCI (Andrews-Hanna et al., 2010; Ishai, 2008). The PCC, a hub of the default mode network (DMN), also showed group differences at the whole-brain level (Table 3 and Figure 3), consistent with prior resting-state studies reporting DMN connectivity loss in MCI (Leveroni et al., 2000; Mirza et al., 2017). Task-based fMRI studies have previously demonstrated that face-related visual tasks induce systematic modulation of the default mode network, particularly in the posterior cingulate cortex, even in neurodegenerative conditions (Sauer et al., 2006). Together, these findings suggest that dysfunction in PHG–PCC circuits underlies early decline in social recognition processes. This result was further supported by an anatomically defined ROI centered at (-18, -34, -8), which showed significant activation overlapping with the group-level cluster (Figure 2), and met peak-level FWE correction within the ROI (Table 8). This convergence strengthens the interpretation of PHG as a reliable region of interest for early detection.

### 4.2 Social cognition networks and preserved behavior

Despite significant neural differences, behavioral accuracy and reaction times were preserved in MCI (Table 2). This dissociation supports the idea that neural dysfunction may precede behavioral decline (Graewe et al., 2013). Prior research has emphasized that social cognition, including face and emotion recognition, is vital for maintaining interpersonal relationships and quality of life (Ramon et al., 2011; Ranganath and Ritchey, 2012). Our results extend these findings by demonstrating that, even when task performance appears intact, fMRI can detect subtle neural deficits in socially meaningful recognition networks. Such neural sensitivity highlights the potential of task-based fMRI as a functional biomarker for early detection of dementia.

### 4.3 Implications for biomarker development

From a biomarker perspective, the identification of early PHG dysfunction is notable (Andrews-Hanna et al., 2010; Ishai, 2008). The ATN framework has emphasized amyloid and tau imaging (Aminoff et al., 2013), but functional changes in social cognition networks may provide complementary information. Recent studies demonstrated that non-verbal and socially meaningful tasks predict disease progression (Canário et al., 2023; Sugiura et al., 2001; Tsukiura et al., 2002). Our results suggest that aberrant activity in PHG and PCC during famous face recognition could serve as a candidate functional biomarker, reflecting the interaction of semantic memory and DMN integrity. Future multimodal studies integrating fMRI with amyloid/tau PET and volumetric MRI are warranted to establish convergent validity. A promising direction for future work is to integrate behavioral markers of cultural and gender-modulated face perception with task-based fMRI. Previous findings in healthy Japanese populations indicate robust cultural and demographic influences on emotion decoding (Hama and Koeda, 2023). Combining these behavioral indices with neural measures may help dissociate disease-specific impairments from culturally shaped perceptual strategies in MCI.

### 4.4 Limitations

Several limitations must be acknowledged. First, the sample size was modest (20 HC and 12 MCI), which may limit statistical power. Second, although groups did not differ significantly in demographics, residual differences in age or education cannot be ruled out. Third, variability in the number of acquired fMRI volumes across participants (155 vs. shorter runs) may have introduced noise, although preprocessing and GLM modeling minimized this issue. Fourth, although all patients met established clinical criteria for MCI, we did not obtain amyloid or tau biomarkers and did not have longitudinal follow-up to determine conversion to Alzheimer’s disease. As such, our findings should not be interpreted as specific to prodromal AD, but rather as reflecting functional alterations associated with the MCI syndrome. Finally, this study focused on famous face recognition; generalization to other social or cognitive tasks requires further work.

## 5 Conclusion

In summary, the present findings suggest that individuals with clinically defined MCI show reduced activation in PHG–PCC networks that support semantic access and internally directed cognition during socially meaningful face processing. Rather than reflecting a deficit specific to famous-face recognition or serving as a direct marker of prodromal Alzheimer’s disease, these alterations may capture early disruptions in PHG–PCC circuitry that normally supports semantic access, internally directed cognition, and integrative processes such as conceptual associations. Such early disruptions in integrative PHG–PCC functioning may represent a neural vulnerability that precedes overt deficits in face-specific social cognition, potentially reflecting one component of the broader processes through which dementia-related changes begin to emerge in some individuals.

## Data Availability

The data supporting the findings of this study are available on OSF at: https://osf.io/H926w/files/osfstorage.
